# A new device-mediated miniprep method

**DOI:** 10.1186/s13568-022-01360-7

**Published:** 2022-02-22

**Authors:** Baryshev Mikhail, Merkulov Dmitrijs, Mironov Ivan

**Affiliations:** 1 Institute of Microbiology and Virology, Riga Stradins University, Ratsupites Str 5, Riga, 1067 Latvia; 2 ELMI Ltd, A Sakharova Str 8-18, Riga, 1021 Latvia

**Keywords:** Plasmid DNA, Miniprep, Supercoiled plasmid, Plasmid DNA isolation

## Abstract

Small-scale plasmid DNA preparation or miniprep is a fundamental technique in estimation cloning experiments and is widely used for DNA methylation analysis in epigenetic research. Current plasmid DNA minipreps use the alkali-SDS-based method in a three-solution format and require spin column-based purification steps. This procedure requires the vortexing or pipetting of pelleted bacteria by centrifugation and manual mixing of the solutions. Here, we describe a centrifuge/mixer-based instrument with the ability to perform centrifugation, vibration, and rotor oscillation in order to perform all steps of plasmid DNA isolation by device only. We found that by applying rotor oscillation-driven mixing of solutions added in the lysis and neutralization steps, homogeneous mixing was achieved within 5 s at a rotor oscillation amplitude of 45° and oscillation frequency of 400 ± 30 rpm, yielding the maximal quantity and quality of plasmid DNA. No increase in host chromosome presence purified by this approach occurs for high-copy-number plasmids compared to manually performed miniprep, and indeed, there is a significant decrease in the presence of the chromosomal fraction in low-copy-number plasmids. The supercoiled form of plasmid DNA purified at a rotor oscillation amplitude of 45° does not turn into an open circular (OC) isoform when the plasmid is stored for 1 year at plus four degrees, in contrast to the plasmid purified with rotor oscillation amplitudes of 270°, 180° and 90°. The programmed time-work-efficient protocol of plasmid miniprep installed in the device gives the extreme simplicity of plasmid minipreps speeding up and facilitating the isolation of plasmid DNAs.

**Supplementary Information:**

The online version contains supplementary material available at 10.1186/s13568-022-01360-7.

## Keypoints

- New devise-mediated plasmid miniprep method (DM) performs all mixing steps without operator intervention.
- The DM method produces plasmid DNAs free of the dCCC form and significantly reduces the contamination with genomic DNA in the low-copy-number plasmid.
- DM miniprep plasmids are reliable templates for bisulfite PCR sequencing analysis.

**Supplementary Information:**

The online version contains supplementary material available at 10.1186/s13568-022-01360-7.

## Introduction

Plasmid DNA minipreps are widely used procedures for recombinant plasmid purification to verify resultant constructs to fit the appropriate downstream application. To perform multiple minipreps, the cost-effective and time-efficient systems that provide researchers with the appropriate quantity and quality plasmid DNA obtained with minimal manual handling are a requirement of the laboratories to compete successfully in generating results. The most commonly used procedures include alkali with SDS (Birnboim and Doly 1979), nonionic detergents (Altschuler et al. 1994) zwitterionic detergents (Chowdhury and Akaike 2005), phenol, and phenol/chloroform extraction with MgCl_2_ addition (Cheng et al. 2004; He et al. 1990; Kovalenko et al. 1994). Several approaches using the colony grown on plate agar as starting material for plasmid minipreps instead of liquid bacterial culture have been described (Sato et al. 2012, 2014). The standard alkali SDS column-based method of minipreps relies on the irreversible denaturation of bacterial genomic DNA under high pH and the ability of plasmid DNA to reassociate upon subsequent neutralization. While chromosomal DNA along with some RNA, protein, and cell debris remains in the precipitate, the plasmids are in solution (Jo et al. 1991). Subsequently, other contaminants are removed by the addition of washing solution. The pure plasmid was then eluted in elution buffer. In various modifications, instead of columns, the plasmid may be purified by organic extraction and ethanol precipitation or differential precipitation with a polyethylene glycol/sodium chloride mixture. The final steps of all of these methods include air-drying for 10–15 min or using a vacuum desiccator.

Plasmids are known to exist in enteric cells in different conformational states: supercoiled DNA (DNA I, or covalent closed circular DNA, i.e. CCCDNA), relaxed circular DNA (DNA II, or open circular DNA, i.e. OCDNA) and linear DNA (DNA III) forms (Vinograd and Lebowitz 1966). In many cases, due to local pH extremes in solutions, the anchor base pairs may be lost. This treatment can then cause the plasmid to form incongruent complementary bases or cruciform loops after neutralization, resulting in an irreversibly denatured supercoiled plasmid form (DNA IV, i.e., dCCCDNA) (Diogo et al. 1999), which is an undesirable impurity in the final plasmid preparation. Some studies have been performed to remove this plasmid DNA form with arginine affinity chromatography and by combining denaturation, selective renaturation and aqueous two-phase extraction (Sousa et al. 2009; Frerix et al. 2007).

Although the column-based alkali procedure gives ready-to-use plasmid solution, the procedure demands the vortexing or pipetting of pelleted bacteria by centrifugation and the admixing of solutions manually. Here, we describe the device with an oscillation-driving mixing option for the steps of solution admixing and the ability for pellet resuspension of 12 samples simultaneously. The time-work-efficient protocol of plasmid purification provides the extreme simplicity of plasmid minipreps, reducing the operating time by 40–60% compared to a manually operating column-based approach. The plasmid DNAs obtained by using the device are free of the dCCCDNA plasmid form and presented mostly by the CCCDNA form suitable for basic molecular biology applications, including sequencing and cell transfection, and can form complexes between the amphipathic TAT peptide and plasmid DNAs that can be used for gene delivery in eukaryotic cells (Saleh et al. 2010). In addition, commercially available “classical” plasmid DNA miniprep column-based alkali kits are compatible with the developed device and can be successfully applied using the installed time- work-efficient protocol.

## Materials and methods

### Bacterial strain and DNA plasmids

Invitrogen (Carlsbad, USA), supplied the XL1-blue *Escherichia coli* strain used for plasmid propagation in LB medium. Antibiotics were purchased from Sigma-Aldrich Biochemie GmbH (Hamburg, Germany). The high copy number plasmids used were pcDNA 3.1/His/LacZ 8.6 kb from Invitrogen Corp. (Carlsbad, USA); pEGFP and pET30 vectors Clontech (Palo Alto, USA); and the low copy number pR322 plasmid from our lab. The pCR4-TOPO Invitrogen (Carlsbad, USA) vector was used to clone 585 and 750 bp PCR-generated fragments of *PPARg2* and *Oct4* gene promoter regions that were used in DNA methylation analysis by PCR bisulfite sequencing. The ZymoPURE™ II Plasmid Midiprep Kit was from Zymo Research Corp. (Irvine, USA). The QIAGEN Plasmid Midi Kit was from QIAGEN GmbH (Hilden, Germany).

### Standard column-based plasmid miniprep

Standard column-based plasmid minipreps were performed according to the GeneJET Plasmid Miniprep Kit, ThermoFisher Scientific Inc. (Carlsbad, USA). (https://tools.thermofisher.com/content/sfs/manuals/MAN0012655_GeneJET_Plasmid_Miniprep_UG.pdf).

### DNA quantification

Average concentrations of the plasmid DNA were quantified using the NanoDrop microvolume sample retention system, Thermo Fisher Scientific Inc. (Waltham, MA).

### Plasmid DNAs storage

Plasmid DNAs purified by the DM and SM miniprep methods were stored in a cold room at 4–6 °C.

### Restriction analysis and DNA sequencing

Restriction endonucleases and T4 DNA ligase were obtained from Thermo Fisher Scientific Inc. (Carlsbad, USA), and used with the buffer stocks recommended and provided by the company.

DNA sequencing was performed using the ABI BigDye Terminator Cycle Sequencing Kit v3.1 ThermoFisher Scientific Inc. (Carlsbad, USA) according to the manufacturer’s instructions on a Gene Amp 9700 PCR machine (Carlsbad, USA), and the sequences were detected on an ABI 3130XL Genetic Analyzer, Applied Biosystems (Carlsbad, USA).

### Assessment of host genomic DNA in minipreps by SQ-PCR

To obtain semiquantitative data on the presence of host genomic DNA in plasmid preparation, four 5 × fold serial dilutions of host chromosome [purified with the Wizard gDNA purification kit; Promega Corp. (Madisson, USA)] in the range 0.008–1 ng were used as the reference of host DNA amount in PCRs and compared with amplification of 50 ng plasmid DNAs. Forward, 5′-TTCCCACGGACATGAAGACTACA-3′ and reverse, 5′-ATCCTGCGCACCAATCAACAA-3′ *E. coli* K-12 strain-specific primers were used to amplify a 1.687 bp fragment (Kuhnert et al. 1995). The relative intensity of the band of the genomic DNA amplified from the plasmid DNA minipreps was quantified in relation to the bands of reference diluted samples by image analysis software Science Lab Image Gauge Ver. 4.0, Fujifilm Corp. (Tokyo, Japan).

### Time efficiency

The time efficiency of the device-mediated minipreps (DM) relative to the standard column-based minipreps (SM) was calculated as the ratio of the total SM and DM procedure completion times amplified by 100. T (tot) SM/T (tot) DM × 100 = % time efficiency. Subtracting 100% we will find how much the DM minipreps more time-efficient, in percentage, compared to SM. T (tot) was considered the sum of the handling times, the time needed for sample/solution manipulation by the operator, and the technological times, which were mediated by the working equipment and incubation time according to the appropriate protocols.

### PC3 cell line transfection

Prostate cancer-derived PC3 cells were cultured in RPMI-1640 culture medium supplemented with 10% fetal bovine serum [Invitrogen Corp. (Carlsbad, USA)] penicillin (100 U/mL), and streptomycin (100 μg/mL) [Invitrogen Corp. (Carlsbad, USA)] at 37 °C in a 5% CO_2_ atmosphere incubator. The cells were seeded in 24-well plates at a density of 1.8 × 10^5^ per well one day before transfection to obtain 80% confluence. Before transfection, the growth medium was removed, and 350 μL of Opti-MEM reduced serum medium [Invitrogen Corp. (Carlsbad, USA)] was added. The K4^®^ Transfection System consisting of the K4^®^ Transfection Reagent and the K4^®^ Multiplier were obtained from [Biontex Laboratories GmbH (München, Germany)] and used according to the company-provided protocol. The transfection efficiency was determined after 24 h of transfection as the percentage of green cells compared to the total cell number.

### Gel retardation assay

One microgram of pcDNA 3.1/His/LacZ plasmid was premixed with various amounts of the GFP-TAT fusion protein (0, 2.5, 5, 7.5, 10, and 20 µg) in phosphate-buffered saline (PBS) to a final volume of 20 µl and incubated at 37 °C for 30 min to form peptide/DNA complexes. *GFP-TAT* fusion was created by fusion PCR and cloned into an IPTG-inducible pET30 vector. The fusion protein was expressed in *E. coli,* and pure protein was subsequently purified by Ni–NTA chromatography. These complexes were analyzed by electrophoresis on a 1% agarose gel in TAE buffer, followed by staining with ethidium bromide.

## Results

### Instrument for devise-mediated miniprep

**Fig. 1:**
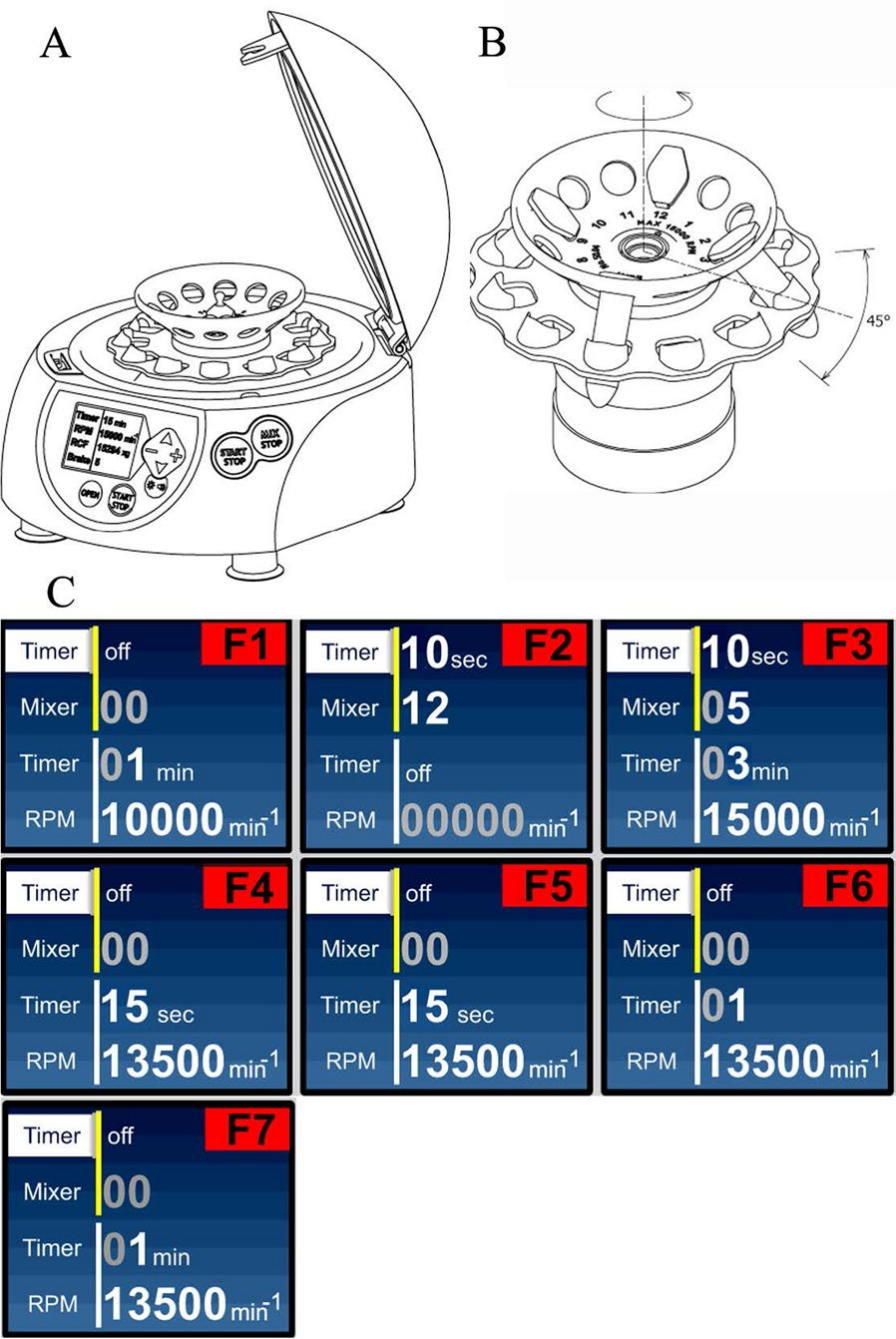
Schematic view of a miniprep assisting instrument assembly.** A** Centrifuge/mixer-based apparatus. **B** Two-part rotor. **C** F1-F7—steps of the DM miniprep protocol. Pre-set parameters for each step of device-mediated miniprep showing the different settings of parameters such as time and speed of centrifugation (F1, F3–F7), time and intensity of vibration for pellet resuspension (F2) and oscillation frequency of rotor motion at amplitude 45° for liquid mixing (F3). F3 is a step that combines the mixing of the solution by oscillation of the rotor with an oscillation frequency of 400 rpm (Mixer 5) and an amplitude of 45° for 10 s, after which the device automatically starts centrifugation. Amplitude of 45° for liquid mixing is a constant parameter in the DM miniprep

The instrument for device-mediated minipreps was created on the basis of an ELMI centrifuge/mixer and has a capacity of up to 12 samples (Fig. 1A). In this study, a special two-part rotor was designed and constructed to perform all steps of the miniprep with the constructed device only (Fig. 1B). The upper part of the rotor comprises the angle disk with openings, while the lower part is the disk with specially shaped openings whose axes lie in one plane with the openings of the upper part of the rotor. This feature of the rotor drives concussion of tubes in the openings of the lower part rotor under rotor vibration, which, in turn, results in quick resuspension of bacterial pellets. The introduced option of back-and-forth rotor movement performs the oscillation-driving mixing of solutions added without tube inversion.

### Plasmid yield by the DM miniprep depends on oscillation-driving mixing parameters and exceeds that by the SM method

The mixing of miscible fluids of different densities must be done within the miniprep procedure. Mixing, as a degree of homogeneity of two or more liquids, plays a pivotal role in the quality and quantity of the final product. In miniprep, it is conventionally carried out by tube inversion. This step is operator-dependent, and some variability in the homogeneity of mixing might occur, which may have an effect on the yield and quality of plasmid DNA. Unlike commonly used 1.5 ml tubes, a 2 ml tube has a cylinder-like geometry and suite to be used in rotor oscillation-driving mixing and will promote an increase in plasmid yield.

**Fig. 2:**
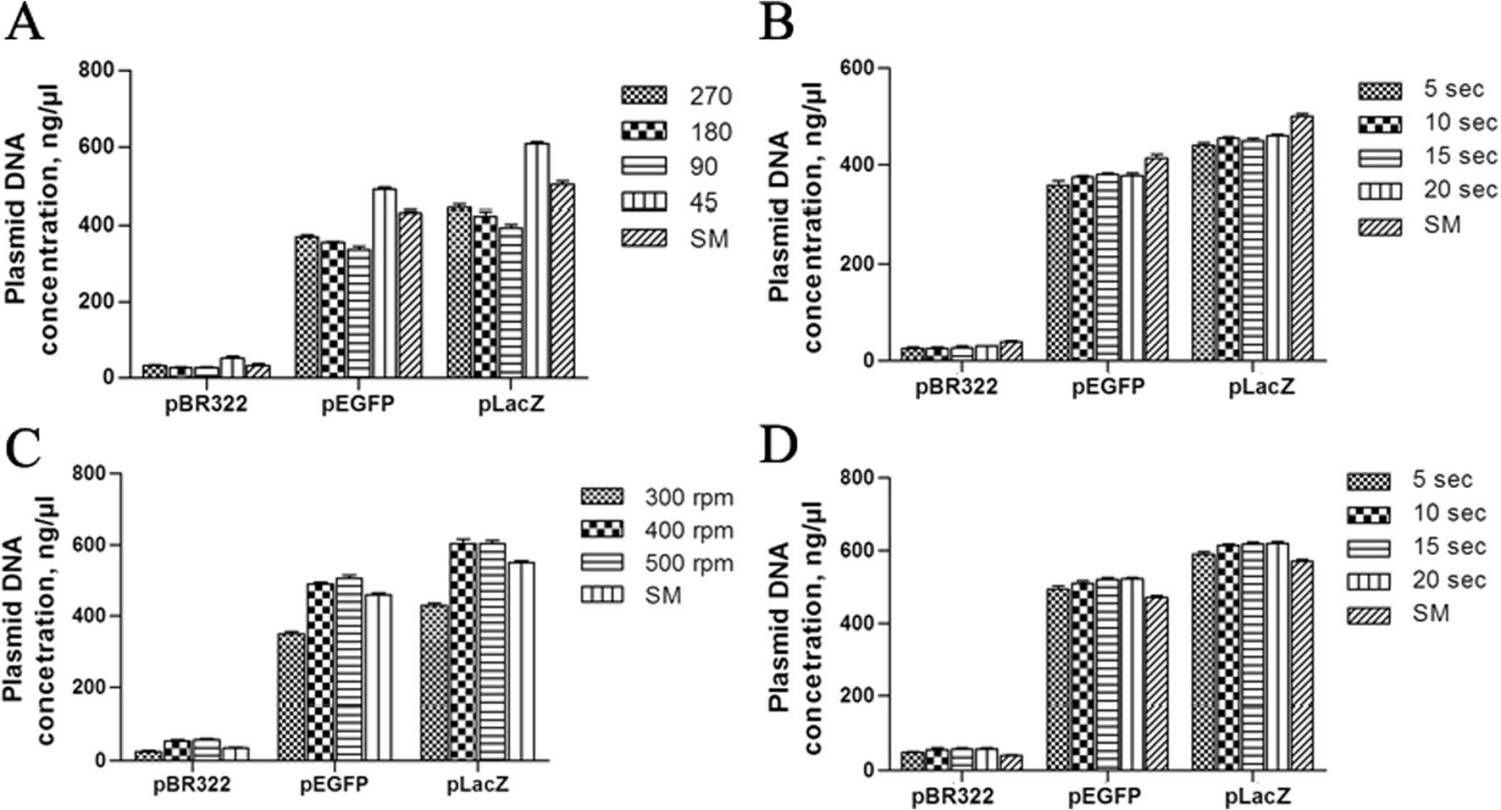
Admixing efficiency of solutions performed by rotor oscillation affects the plasmid DNA yields. **A** Effect of oscillation amplitude of 5 s of oscillation-driving mixing in DM minipreps at an oscillation frequency of 400 rpm on plasmid yields. **B** Effect of time oscillation driving mixing in DM minipreps at oscillation amplitude 270° and oscillation frequency 400 rpm on plasmid yields. **C** Effect of oscillation frequency of 5 s of oscillation driving mixing in DM minipreps at oscillation amplitude 45° on plasmid yields. **D** Effect of time oscillation driving mixing at oscillation amplitude 45° and oscillation frequency 400 rpm in DM minipreps on plasmid yields. Assays were performed in triplicate, and error bars represent the standard deviation

**Fig. 3:**
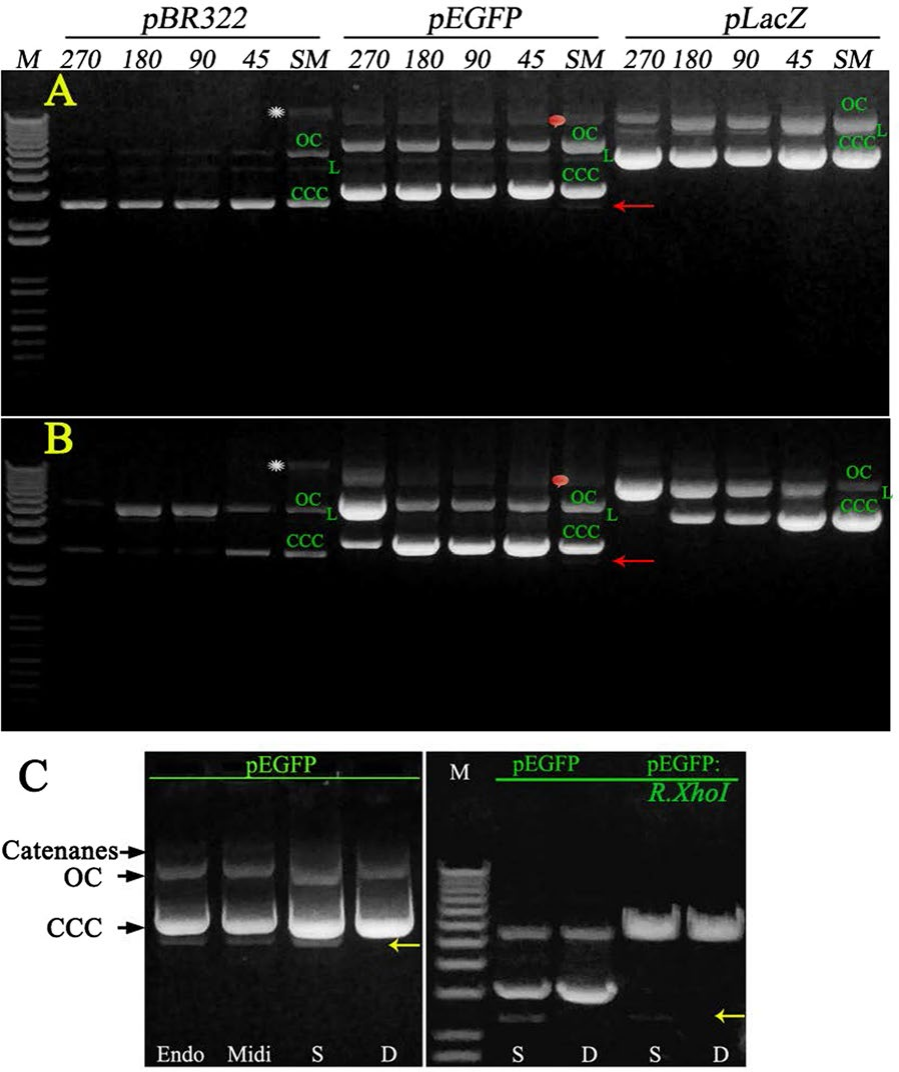
DM minipreps plasmids are purer compared to SM plasmids. **A** Comparison of DM and SM minipreps plasmid conformational states. DM, SM miniprep plasmids were eluted in 50 µl of elution buffer. 1 µl of the eluted high and 5 µl low copy number plasmid were loaded in the well in a 15 µl total volume. Plasmids were subjected to electrophoresis on 0.8% agarose gel. Migration of CCC, OC and linear (L) forms of plasmid DNA is indicated by the green abbreviation in the corresponding band. The red pictogram shows the migration of catenated forms of pEGFP. The presence of host genomic DNA in SM-isolated pBR322 is depicted by an asterisk. **B** Comparison of the DNA stability of DM and SM miniprep plasmids after one year of storage at + 4°. DNA sample loading and legend are the same as shown in **A**. **C** Comparison of pEGFP of DM (D) and SM (S) minipreps plasmid quality and capability of cleavage by restriction endonuclease in comparison to ZymoPURE-EndoZero Midiprep (Endo) and QIAGEN Midi Kits (Midi) isolated plasmid. 1 µl of pEGFP by SM and DM miniprtepped samples were digested with 1 µl of *XhoI* at 37 °C for 1 h in 15 µl of the recommended buffer. The plasmid digestion products were loaded into wells and separated by electrophoresis on a 0.8% agarose gel. The 1 kb plus DNA ladder was electrophoresed as a DNA size marker. dCCCDNA is indicated by the arrowhead and is resistant to restriction enzyme digestion

We introduced oscillation-driven mixing to avoid manual operation and to make the process of plasmid DNA isolation ‘semi-automated’. The oscillating motion of the rotor provides oppositely vectored acceleration forces, which are automatically imposed on the constituent of the tube to mix the solutions. To ensure proper mixing, the tube is periodically oscillated in opposite directions so that the various formed components will gravitate first toward one end of the tube wall and then toward the opposite end of the tube wall. We compared the effect of oscillation-driven mixing at various amplitudes and frequencies on plasmid DNA yield (Fig. 2A–D) and found that the process of mixing is time-, oscillation amplitude- and oscillation frequency-dependent and affects plasmid quality and yield. Having evaluated parameters affecting plasmid yield by this approach, we found that a combination of oscillation amplitude 45° and oscillation frequency 400 rpm ensures the homogenous mixing of the admixed solutions during minipreps in 5–10 s, yielding the amount of plasmid DNA exceeding that of the manually isolated plasmid DNA (Fig. 2A, C, D). In addition to a higher plasmid yield, an improvement in the quality of the plasmid was also observed: the pEGFP plasmid was free of the dCCCDNA plasmid form (Fig. 3A, C). It is interesting to note that the plasmid yield at a rotor oscillation amplitude of 90° is less than that obtained at 270° and 180°. Furthermore, the plasmid yield at these three amplitudes did not reach that obtained by the SM method even after 20 s of mixing (Fig. 2A, B), reflecting incomplete/improper mixing. In general, the mixing efficiency is increased upon decreasing the amplitude oscillation but not in the case of 90°. However, the mixing performance at an amplitude of 45°, which is half of 90°, and an oscillation frequency of 400 rpm provides a homogenously mixed solution in 5–10 s, resulting in an excessive plasmid yield compared to the SM method (Fig. 2A, D). Moreover, plasmids purified under these conditions retained supercoiling after storage for one year at + 4°, in contrast to the plasmids isolated with rotor oscillation amplitudes of 270°, 180° and 90° (Fig. 3B**)**.

Of note, the low copy number plasmid prepared demonstrated the absence of visible chromosomal impurity on the gel, in contrast to the manually prepared plasmid (Fig. 3A, B). Having determined the mixing parameters giving improved miniprep, we established the DM miniprep and used it in the plasmid purification.

### Devise-mediated miniprep

Miniprep consists of seven preprogrammed successive steps using the GeneJET column-based plasmid miniprep kit. The preset optimized parameters for each miniprep step are installed in the device and represent the DM miniprep protocol, as shown in Fig. 1C. Of what you need to perform miniprep is to add/remove solutions and press the start button of the following step to obtain the ready for use plasmid DNA in the final step.

- F1. Spin 2 ml of overnight grown bacterial culture. Remove the supernatant.
- F2. Add 250 μl of resuspension solution. Press Start. All pellets will be resuspended.
- F3. Add 250 μl of lysis solution and then add 350 μl of neutralization solution. Press Start. All solutions will be mixed and automatically centrifuged, removing the main impurity.
- F4. The supernatant was poured into a column/tube ensemble. Press Start. Discard the flow throw.
- F5. Add the washing solution. Press Start. Discard the flow throw.
- F6. Place tubes into the device. Press Start. The remaining washing solution will be removed.
- F7. Add 50 μl of elution buffer and put the tubes into the device for 1 min. Having increased the temperature within the device during operation will promote plasmid DNA elution. In a 1-min press Start. Eluted plasmid DNAs are ready to use.

Purified by this protocol, plasmids had OD260/280 in a range of 1.85–1.95, which indicates that the plasmids are pure enough to be tested in plasmid quality-sensitive downstream applications. The average yields of the plasmids obtained were 3 ± 0.5 µg for pBR322, 22 ± 4 µg for pEGFP, and 30 ± 3 µg for pLacZ. The pLacZ plasmid yield from 2 ml overnight *E. coli* culture reached up to 30 µg, which corresponds to 3 mg of the plasmid being isolated from 200 ml bacterial culture by DM miniprep.

### Devise-mediated minipreps are time efficient compared to standard column-based plasmid minipreps

The steps of miniprep performed by the newly developed device use the different settings of parameters such as the time and speed of centrifugation, the intensity of vibration used for pellet resuspension and the oscillation amplitude and oscillation frequency of rotor motion for liquid mixing. To minimize the miniprep time, the optimized miniprep procedure parameter settings were set up and installed in the device. Optimization ensures the completeness of the cell resuspension, bacterial lysis and solution mixing needed for miniprep performance. Figure 1C shows the parameter settings for each step. After the step is complete, the device automatically goes to the next step highlighting it on the screen. To evaluate the time efficiency, we compared the DM miniprep protocol versus the SM protocol. The data presented in Table 1 illustrate the time efficiency of DM minipreps. The plasmid isolation using DM minipreps saves time up to 60% upon processing 12 samples regarding the SM miniprep and requires 20 min to complete the procedure (Table 1). It should be noted that the recently published one-step miniprep method accomplishes one sample only for 26 min, excluding the time for plasmid drying and dissolution (Lezin et al. 2011). Our DM miniprep excludes the manual handling of solution mixing and cell pellet resuspension and decreases the total time of the minipreps procedure, making plasmid isolation faster than ever.

**Table 1:** Time efficiency of devise-mediated vs standard minipreps

	Devise-mediated MiniPrep		Standard MiniPrep		
Number of preps	Total time, min	Operator’ time	Total time min	Operator’ time	Time Efficiency, %
2	12	4	17	5	42
4	14	7	21	9	50
8	17	10	26	14	53
12	20	13	32	20	60

### The DM plasmid is pure for restriction digestion and sequencing, is free of the dCCC DNA plasmid form and has a lower amount of host genomic DNA

We compared the sensitivity of the plasmid DNA purified by either DM or SM minipreps in restriction enzyme digestion and sequencing reactions. Although DM and SM minipreps produced similar amounts of plasmid DNA, we observed a sustainable tendency in increasing the yield and improving the quality of plasmid DNA in the sense of plasmid topological structure and the presence of chromosomal fraction among the three plasmids isolated by DM miniprep. In the pEGFP plasmid, which is often used in cell transfection experiments, we did not observe the dCCCDNA plasmid form or some chromosomal fraction visible in SM pBR322 (Fig. 3A–C).

**Fig. 4:**
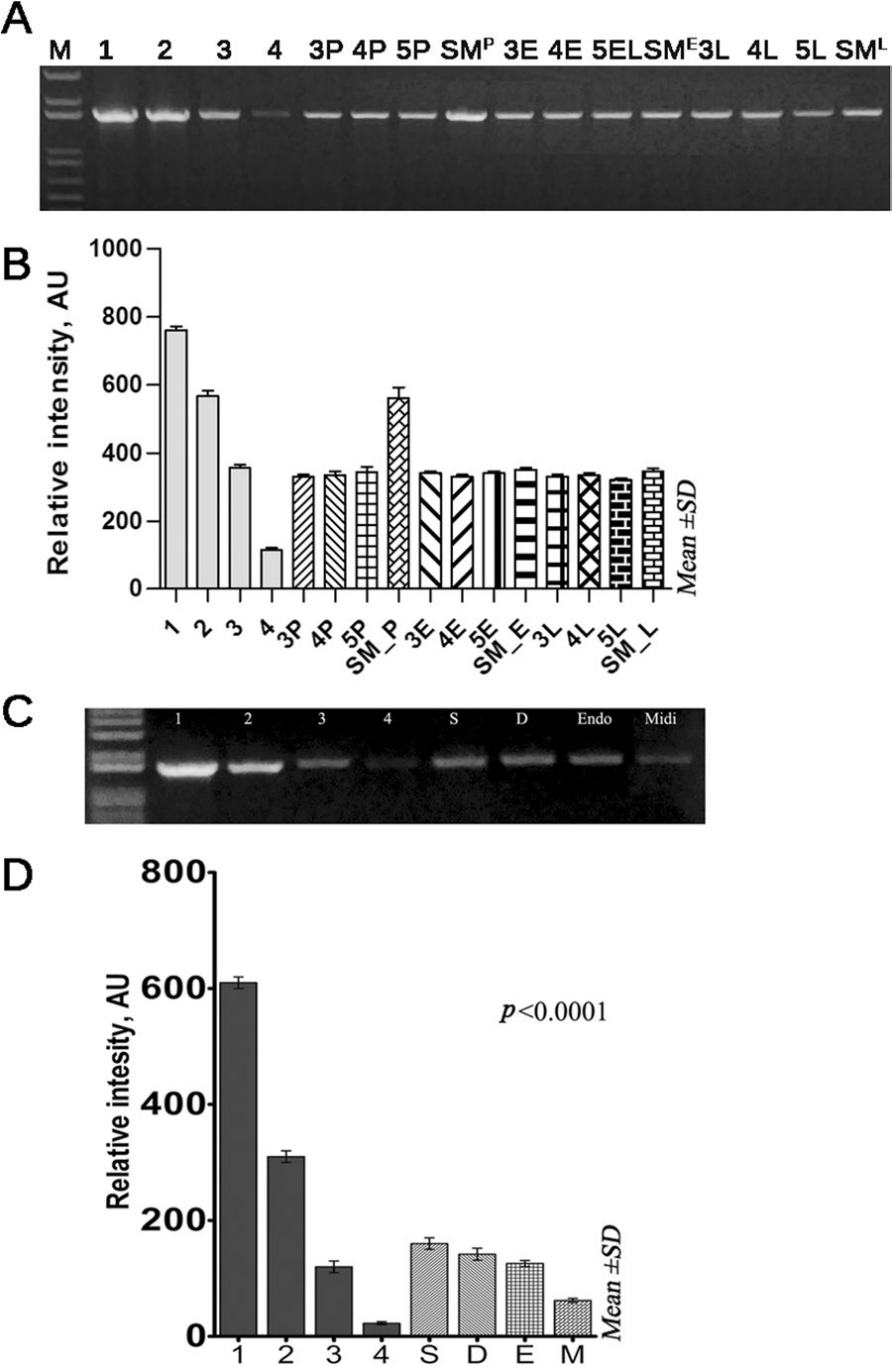
DM minipreps plasmids have a lower amount of host genomic DNA. **A** The amount of host genomic DNA in DM and SM plasmids; lanes 1–4—reference interval of quantities’ DNA for semiquantitative analysis; P, E, L—pBR322, pEGFP, LacZ plasmids purified by DM at oscillation amplitude 45° and 3, 4, 5—300, 400, 500 rpm oscillation frequency; SM^P^, SM^E^, SM^L^—pBR322, pEGFP, LacZ plasmids purified by SM method. **B** Densitometry analysis of amplified products is presented. **C** The amount of host genomic DNA in pEGFP plasmid DNA purified either by SM (S) or DM (D) minipreps and the ZymoPURE-EndoZero (Endo) Midiprep or QIAGEN Midi kit (Midi). **D** Densitometry analysis of amplified products is presented. Assays were performed in triplicate, and error bars represent the standard deviation

We also found that DM miniprep plasmid DNA is a robust template in sequencing reactions of *PPARg2* and *Oct4* promotor region fragments of 585 and 750 bp in size, respectively, representing the bisulphite-treated inserts used in DNA methylation analysis of the genes (Additional file 1: Fig. S1, S2). All plasmids used in the analysis of DNA methylation of distal and proximal *Oct4* enhancers (Baryshev et al. 2018) and the proximal *PPARg2* promoter (Baryshev et al. 2022) were obtained by the DM miniprep method. We compared the presence of host genomic DNA in the plasmids obtained by DM, SM minipreps and commercial non-miniprep kits recommended for purification of high-quality plasmid DNA. From the data presented in Fig. 4A, B, it can be seen that a small fraction of the host genomic DNA in the range of 100–150 pg per 50 ng of plasmid is present in all purified plasmids, and the DM preparation contains less chromosomal DNA than the SM. According to densitometry analysis, the chromosomal fraction found in the plasmids was 60 ± 5 pg for the QIAGEN Plasmid Midi Kit, 120 ± 10 pg for the ZymoPURE™ II Plasmid Midiprep Kit, 105 ± 10 for the DM miniprep and 130 ± 10 for the SM miniprep (Fig. 4C, D). We further revealed that the use of DM mixing options has a great advantage for low copy number plasmid pBR322 production compared to the SM method. Any of the DM mixing parameters studied are able to produce a predominantly monomeric CCC form of the plasmid, avoiding the presence of a visible chromosomal fraction on the gel, in contrast to the SM method (Fig. 3A, B). Meanwhile, we did not observe the same effect for high-copy-number plasmids.

Thus, our results show that the hydrodynamic forces arising at a rotor oscillation frequency specified do not affect the chromosomal shearing in DM miniprep and that the content of host genomic DNA in the final DM plasmid preparation is sustainably lower than that in the SM isolated plasmid (Fig. 4A–D).

### The DM pEGFP plasmid has similar transfection efficiency compared to the EndoZero and Midi kit purified plasmid

**Fig. 5:**
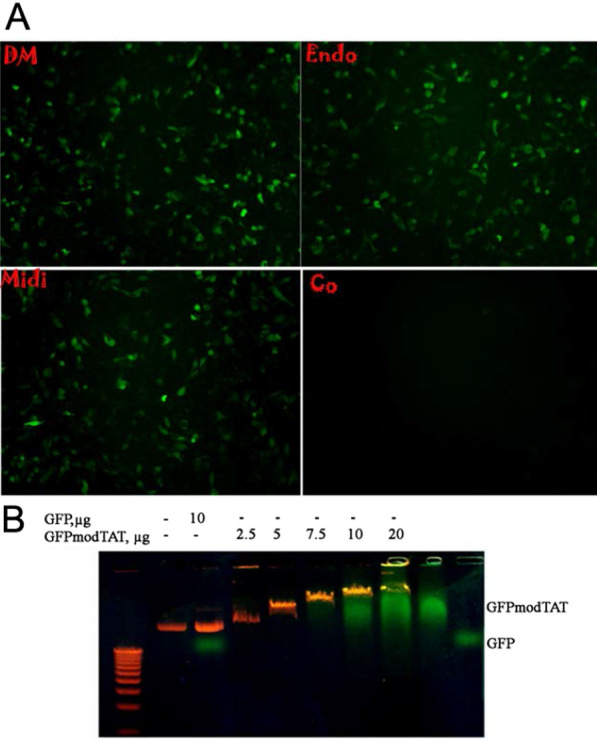
DM miniprep plasmids can efficiently be used in DNA quality-sensitive applications. **A** The transfection efficiency of PC3 prostate cancer cells with the DM (DM) minipreps plasmid was similar to that of the ZymoPURE-EndoZero Midiprep (Endo) and QIAGEN Midi (Midi) Kit-derived results. One of three independent experiments is reported. **B** pcDNA 3.1/His/LacZ plasmid purified with the DM minipreps method forms GFP-TAT-fusion protein complexes. pcDNA 3.1/His/LacZ plasmid was premixed with various amounts of the GFP-TAT fusion protein as described in methods. Samples were resolved by agarose gel electrophoresis. GFPmodTAT and GFP written on the right correspond to pure proteins.

The transfection efficiency of the pEGFP plasmid purified by the DM approach was compared with that of the same plasmid purified with commercial EndoZero and Quiagen Midi kits. The results presented in Fig. 5A show that plasmids purified by DM miniprep were able to transfect 35% of PC3 cells, whereas EndoZero and Quiagen Midi kits purified plasmids transfected 34% and 33% of cells, respectively. Similar transfection efficiency was observed among the three plasmids tested, suggesting that DM miniprep plasmid DNA can successfully be used in cell transfection experiments, making these experiments more cost- and time-effective.

### GFP-TAT binding

To test whether the GFP-TAT fusion protein could bind to DNA in vitro, the pcDNA 3.1/His/LacZ plasmid was mixed with various concentrations of GFP-TAT fusion. The formed complexes were analyzed by electrophoresis on an agarose gel and stained with ethidium bromide (Fig. 5B). As seen in the figure, the migration of supercoiled DNA bands is shifted with increasing amounts of GFP-TAT fusion. These results suggested that the positively charged TAT peptide might bind to plasmid DNA. This binding would occur between the negative charges of plasmid DNA and the positive charges of the TAT protein transduction domain of the fusion protein via electrostatic interactions.

## Discussion

Although plasmid DNA purification is a well-established method in molecular biology, the technique is still developing for time-, cost saving and decreasing labor, especially upon processing multiple samples. It was found that with a decrease in the time and speed of centrifugation of the bacterial culture, the time for resuspending the bacterial sediment using a vortex device is significantly reduced (Voo and Jacobsen 1998). The plasmid DNA recovery in miniprep occurs by alkaline cell lysis followed by neutralization with an acidic high molar potassium acetate. After neutralization, the cell debris, high molecular weight chromosomal DNA and other impurities become trapped within the soft, buoyant and highly shear-sensitive gel matrix (Ciccolini et al. 1999). The separation of the plasmid-containing solution from the floating gel matrix was carried out by high-speed centrifugation. The mixing conditions and the alkaline incubation time are critical in plasmid minipreps. Many plasmid miniprep protocols indicate 5-min incubation for the lysis of bacteria that were mixed with lysis buffer by vigorously inverting the tube. Thereafter, neutralization was carried out by adding chilled neutralization buffer and inverting the tube, followed by incubation on ice for 5 min (Qiagen Plasmid Handbook 2021). During the lysis step, intracellular constituents are released within a few seconds, and the chromosomal DNA is irreversibly denatured under extreme alkaline conditions, while plasmid DNA is capable of annealing after the neutralization step. If the alkaline environment during lysis exceeds 0.15 ± 0.03 M (pH 12.9 ± 0.2), irreversible denaturation of the supercoiled plasmid DNA occurs (Meacle et al. 2004). Therefore, when the lysis buffer of 0.2 M NaOH range is mixed with the resuspended cells, some of the cells will experience a locally high pH, leading to plasmid denaturation that is shown to increase with time of exposure, and as a result, denatured supercoiled plasmid DNA appears in the final eluate of SM Miniprep (Fig. 3A–C). Yu et al. (2008) showed that such alkaline denatured supercoiled DNA has a stable conformation with unregistered, topologically constrained double strands and an intrastranded secondary structure. This form is not effective in gene transfer and constitutes important impurities due to their physical and chemical similarities with the supercoiled form (Prazeres et al. 1998). It has been shown that lysis of 4 ml *E. coli* suspension occurs within 30–40 s (Ciccolini et al. 1999) and that the pure supercoiled plasmid denatured rapidly in 0.2 M NaOH (Meacle et al. 2004).

Taking into account these data and our observation that the cell suspension obtained under the conditions of steps 1 and 2 (Fig. 1C) of the DM miniprep method is lysed immediately (becomes clear) after the addition of the lysis solution, we excluded the incubation time in alkali and the mixing as well and added a neutralizing solution at once. This will minimize the exposure of the plasmid to an alkaline environment, its denaturation, and, we assume, will fall off the effect of a shear in genomic DNA. Due to the change in the rheological properties of the lysate during the lysis steps and especially the neutralization step, it is difficult to achieve gentle and efficient mixing even in miniprep format with regard to shearing genomic DNA to avoid the presence of this contaminant in the low copy number plasmid obtained by the standard miniprep method (Fig. 3A). The addition of a denser (d = 1.16 g/cm^3^) neutralizing solution settle it to the bottom of the tube, and to ensure faster renaturation of denatured plasmids, rapid intensive mixing could be a correct strategy. Therefore, we have introduced rotor oscillation-driving mixing in the miniprep procedure. With these modifications, we demonstrated that the mixing efficiency of the solution affects the plasmid quality and yield (Fig. 2). We determined that the combination of oscillation amplitude 45° and oscillation frequency 400 rpm ensures homogenous admixing of solutions during minipreps in 5 s, yielding the amount of plasmid DNA exceeding that of the SM isolated plasmid (Fig. 2A, C, D).

Taking into consideration the hydrodynamic aspects of fluid mixing at a relatively high rotor oscillation frequency, which can lead to a force generated by the liquid due to turbulence and might have an effect on chromosomal shearing, we assessed the presence of the host genomic DNA in plasmid preparations (Fig. 4). Unexpectedly, our results show that the action of fluid hydrodynamic stresses caused by the movement of the rotor with the indicated oscillation frequency does not affect chromosomal shearing in the DM miniprep, and the content of the host genomic DNA in the final plasmid preparation is consistently lower than that in the isolated SM plasmid (Fig. 4A–D). This is consistent with the finding that efficient mixing during neutralization achieved with a Rushton turbine and agitation speed in the range of 190–1200 rpm does not lead to contamination of the plasmid DNA with the chromosomal fraction (Chamsart et al. 2001). However, the isolated plasmid DNAs studied are mainly found in the form of OC, even with gentle mixing. It seems that prolonged incubation in alkali and a 5-min neutralization step promote the turning of CCC to the OC isomer of the plasmid. In addition, we provide evidence that the presence of host genomic DNA is observed even when using a plasmid isolation kit other than the miniprep format. From the data presented in Fig. 4C, D, one can conclude that a small fraction of host genomic DNA in the range of 60–150 pg per 50 ng of plasmid, representing an impurity of plasmid preparations and detected in plasmid samples obtained by both DM, SM minipreps and commercial non-miniprep kits, using alkaline lysis of bacterial cells, arises due to the shearing of the host chromosome during alkaline lysis of cells. Improving the selective CCCDNA plasmid/chromosomal DNA binding/elution conditions to silica membranes can help avoid plasmid preparation to contain the host genomic DNA fraction.

Surprisingly, all plasmids purified by the DM method and stored at + 4° for one year showed differences in the stability of CCC form plasmid DNA (Fig. 3B). If plasmids obtained by the SM and DM (oscillation amplitude 45°, oscillation frequency 400 rpm) methods retained the CCC form as predominant, then supercoiling of high copy number plasmids isolated by DM at 270° and low copy number plasmids isolated at 180 and 90° of mixing options was almost completely lost, turning into an OC isoform (Fig. 3B). In *E. coli,* the topological state of plasmid DNA is under the control of four DNA topoisomerases that are able to alter negative supercoils from the CCC form of DNA (Higgins and Vologodskii 2015). It seems that the observation of changes in the topological behavior of plasmids that we observe after long-term storage is mediated by the copurification of Topo I/Topo III of type I enzymes, which break one strand at a time, converting the supercoiled form to the OC isoform. Thus, the DM method can differentiate plasmid isolation relative to possible copurification of the CCC plasmid-Topo I/Topo III complexes and may be of interest for supercoiling studies.

Here, we have introduced the new DM miniprep method. We assume that an increase in the yield of plasmid DNA and their higher quality achieved with DM miniprep is associated with a short exposure time to an alkaline environment, faster step of neutralization of minipreps, and more efficient mixing of solutions using rotor oscillations. The plasmid DNAs purified using the DM method and the GeneJET miniprep kit are free of the dCCCDNA plasmid topological invariant and contain less genomic DNA. We expanded the field of application of DM miniprep plasmids and showed that the quality of the plasmids is high enough to be used in studies of protein/peptide-DNA binding and that plasmids purified by the DM method can be widely used in DNA methylation analysis. Our DM method can differentiate plasmid isolation relative to possible copurification of the CCC plasmid-Topo I/Topo III complexes and may be of interest for supercoiling studies. With the DM method, miniprep is a less labor procedure for plasmid miniprep with minimal manipulation and can be considered a “semiautomatic” method, allowing up to 60% timesaving when processing 12 samples compared to SM. The above approach can be especially useful for researchers, who have limited access to laboratory supplies and equipment and require a rapid method and apparatus for isolating plasmid DNA.

## Supplementary Information

**Additional file 1: Fig. S1.** Sequencing of DM miniprepped DNA applied for mouse *PPARg2 *DNA methylation analysis. **Fig. S2.** Sequencing of DM miniprepped DNA applied for human *Oct4 *promoter DNA methylation analysis.

## Data Availability

The data generated and analyzed during the current study are available from the corresponding author on reasonable request.
