# Supplementary material for: A new device-mediated miniprep method
Source: AMB Express. 2022 Feb 22;12:21. doi: 10.1186/s13568-022-01360-7 (PMC8863996; doi:10.1186/s13568-022-01360-7)

Supplementary material

AMB Express

A new device-mediated miniprep method

Baryshev Mikhail[1]*, Merkulov Dmitrijs[2], Mironov Ivan[2]

1 Institute of Microbiology and Virology, Riga Stradins University, Ratsupites Str 5, LV-1067, Riga, Latvia;

2 ELMI Ltd, A Sakharova Str 8-18, LV-1021, Riga, Latvia.

*Corresponding author: E-mail: Mihails.Barisevs@rsu.lv

E-mail: **Mihails.Barisevs@rsu.lv**; Telephone +37128280853

Supplementary figures

Supplementary Fig. S1 Sequencing of DM miniprepped DNA applied for mouse *PPARg2* DNA methylation analysis.

File: GEC9883_2019-11-12_1573573083182.ab1 | Run Ended: Nov 12, 2019, 17:37:49 | Signal G:550 A:479 T:491 C:88 | Comment:
Sample: GEC9883 | Lane: 13 Base spacing 12.88 | 989 bases in 13811 scans |

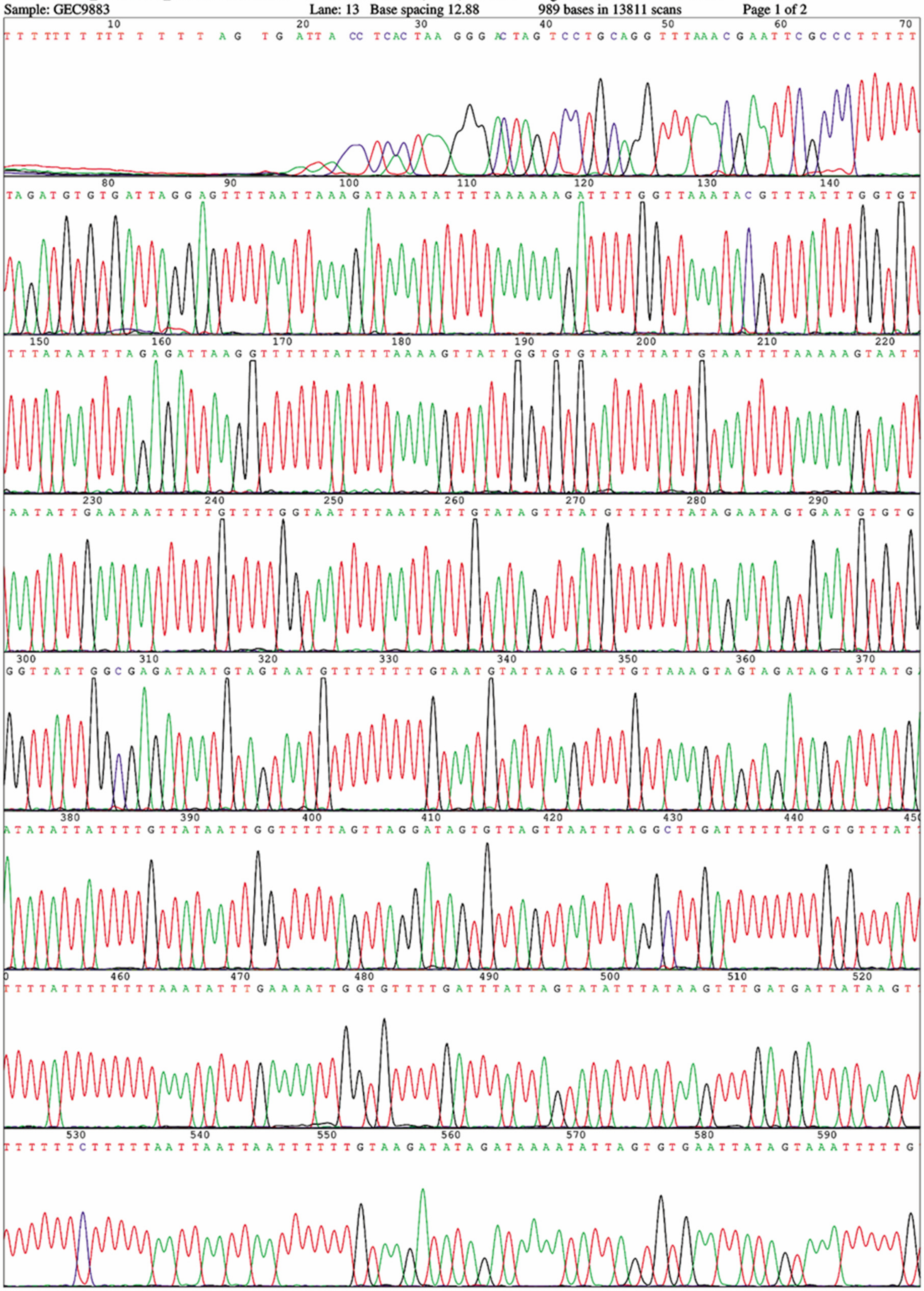

Supplementary Fig. S2 Sequencing of DM miniprepped DNA applied for human *Oct4* promoter DNA methylation analysis.

File: anh3_2014-02-13_1392299388484.ab1 Run Ended: Feb 13, 2014, 15:49:38 Signal C:112 T:475 A:445 G:372 Comment:
Sample: anh3 Lane: 2 Base spacing 18.41 597 bases in 7094 scans Page 1 of 1

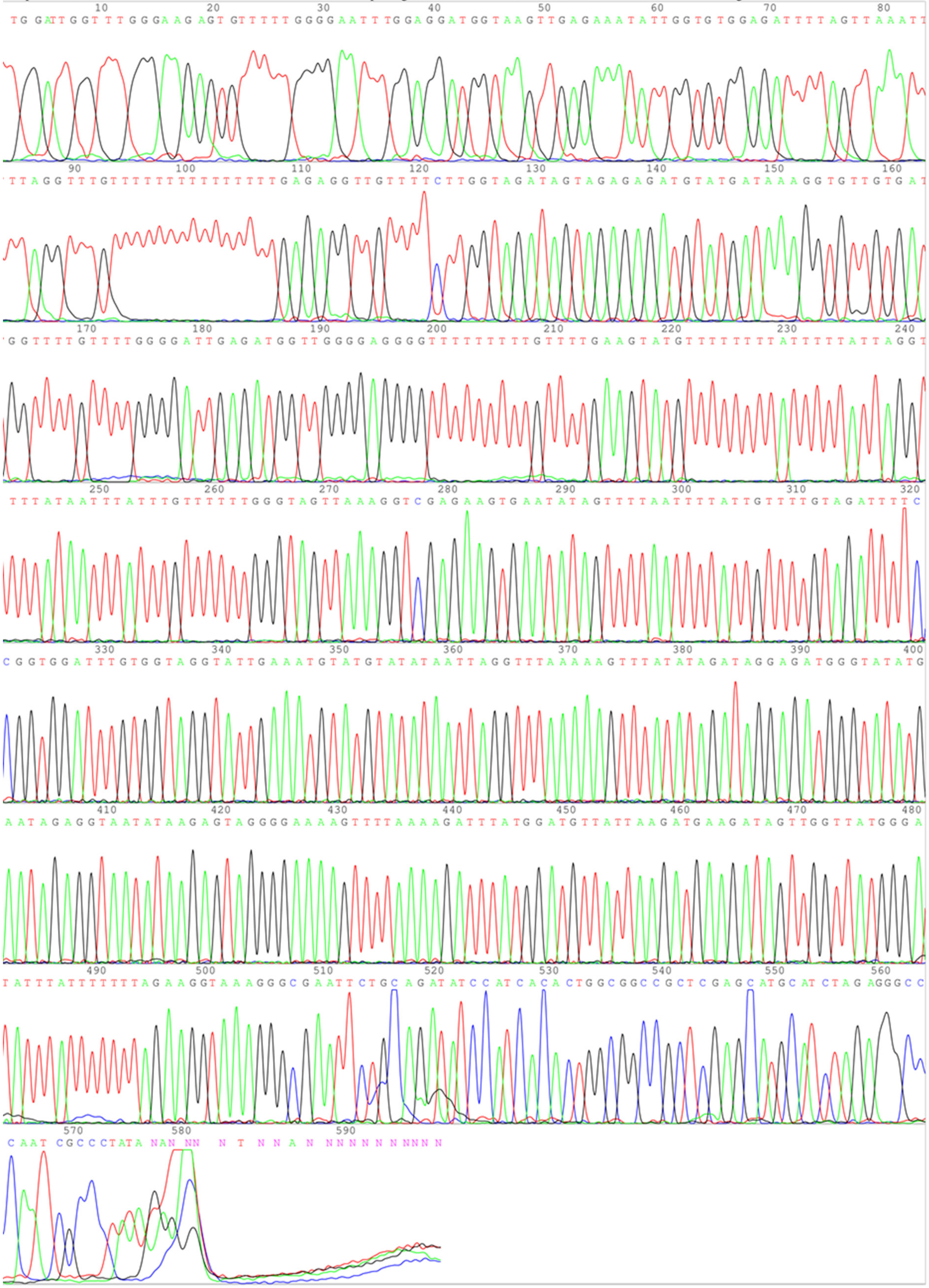

Supplement: Supplementary file 1 — Additional file 1: Fig. S1. Sequencing of DM miniprepped DNA applied for mouse PPARg2 DNA methylation analysis. Fig. S2. Sequencing of DM miniprepped DNA applied for human Oct4 promoter DNA methylation analysis. [file 13568_2022_1360_MOESM1_ESM.pdf]
